# Free word association analysis of German laypeople’s perception of biodiversity and its loss

**DOI:** 10.3389/fpsyg.2023.1112182

**Published:** 2023-06-28

**Authors:** Annike Eylering, Kerstin Neufeld, Felix Kottmann, Sebastian Holt, Florian Fiebelkorn

**Affiliations:** Biology Didactics, Department of Biology and Chemistry, Osnabrück University, Osnabrück, Germany

**Keywords:** social representation theory, biodiversity crisis, free word association test, network analysis, climate change, species loss, IUCN red list of threatened species

## Abstract

Due to the dramatic biodiversity crisis, it is crucial to understand how people perceive biodiversity. Knowledge of how thoughts are organized around this concept can identify which ideas are best to focus on biodiversity conservation information campaigns. The primary aim of the present study was to identify social representations of the German public regarding the concept of biodiversity and its loss using a free word association test. Furthermore, unique association networks were analyzed. For this purpose, data collection was performed in September 2021 in Germany using an online questionnaire to assess participants’ associations with the prompt “biodiversity” (*n*  = 131) and “biodiversity loss” (*n*  = 130). Additionally, we used the social network software Gephi to create biodiversity (loss) association networks. The five most commonly mentioned associations for biodiversity were “animal,” “plant,” “nature,” “human,” and “flower.” For biodiversity loss, the five most commonly mentioned associations were “species extinction,” “climate change,” “plant,” “insect,” and “bee.” Neither “land use change” nor “invasive species,” as key drivers of biodiversity loss, were present in social representations of the German public. A difference was observed in the total number of mentioned associations between biodiversity and biodiversity loss. For both, the associations “plant” and “animal” were related. However, participants associated specific taxa only with animals, such as “insects” and “birds.” For plants, no specific taxa were named. Based on the network analysis, the most commonly mentioned word pairs for biodiversity and biodiversity loss were “plant – animal” and “species loss – climate change,” respectively. Based on our statistical network analysis, these associations were identified as the most central associations with the greatest influence in the network. Thus, they had the most connections and the function of predicting the flow in the network. In sum, the public’s multifaceted views on biodiversity and its loss, as well as the aforementioned central associations, hold great potential to be utilized more for the communication and education of biodiversity conservation. In addition, our findings contribute to the scientific community’s understanding of social representations and perceptions of biodiversity and its loss.

## 1. Introduction

Biological diversity, which is synonymous with “biodiversity” (Menzel and Bögeholz, 2009), is the scientific term used for the variety of life on Earth (Chivian and Bernstein, 2010). Biodiversity is regarded as a multidimensional, abstract, and complex concept (van Weelie and Wals, 2002; Zemits, 2006; Dikmenli, 2010). Precisely because biodiversity is such an abstract concept and can be interpreted in various ways, it is difficult to communicate biodiversity issues to the public (van Weelie and Wals, 2002). For example, according to the Convention on Biological Diversity (CBD), biodiversity can be defined as “*the variability among living organisms from all sources, including terrestrial, marine and other aquatic ecosystems and their ecological complexes, encompassing diversity within species, between species and within ecosystems*” (United Nations Conference on Environment and Development (UN), 1992, Article 2).

The concept of biodiversity has many shapes and forms. Apart from the ecological dimension, biodiversity encompasses social, ethical, and economic dimensions (Gayford, 2000). For instance, in sustainability policy, biodiversity is considered a natural resource; however, in evolutionary theory, it is considered a product of evolution (van Weelie and Wals, 2002). In the present study, the working definition of “biodiversity” is based on three levels: (1) genetic diversity, which depends on heritable variation within and between populations of organisms; (2) species diversity, which describes the number of species in each area; (3) ecosystem diversity, which can be inferred with the help of species diversity, since it increases with increasing ecosystem diversity (Swingland, 2001).

Biodiversity is the primary indicator of ecosystem health. Notably, humans cannot live without the ecosystem services that biodiversity offers (Chivian and Bernstein, 2010). This reliance implies that healthy ecosystems clean our water, purify our air, maintain our soil, regulate the climate, recycle nutrients, and provide us with food. Moreover, healthy ecosystems provide raw materials and resources for medicines and serve additional purposes, such as—being the foundation of all civilization and sustaining global economies (Chivian and Bernstein, 2010; IPBES, 2019). However, biodiversity is declining at an unprecedented rate in human history, which will have severe impacts on people and the environment (IPBES, 2019).

This problem is less known and covered in the media than issues related to climate change (Legagneux et al., 2018). Moreover, there is increasing concern about the consequences of biodiversity loss on ecosystem functioning and the resulting provision of ecosystem services (Balvanera et al., 2006). The main driver of biodiversity loss is human behavior (Saunders et al., 2006). There are three key drivers of biodiversity loss caused by human behavior: (1) climate change; (2) land use change (including sealing of land, fragmentation of landscapes, and alteration of natural water body structures); (3) exposure to nutrients and pollutants (Mohaupt-Jahr and Küchler-Krischun, 2008; IPBES, 2019). The consequences of this environmental degradation affect both our quality of life and economic prosperity (Ehrlich and Ehrlich, 2013; Dirzo et al., 2014; Sockhill et al., 2022) while also leading to irreversible biodiversity loss.

National Biodiversity Strategies and Action Plans (NBSAPs) are instruments used to incorporate biodiversity protection plans and measures in CBD parties [United Nations Conference on Environment and Development (UN), 1992]. Germany implemented a ‘National Strategy on Biological Diversity’ (NBS) in 2007, as well as the ‘Nature Conservation Action Programme 2020’ [Federal Ministry for the Environment, Nature Conservation, Nuclear Safety and Consumer Protection (BMUB), 2007, 2015]. The strategy describes fields of action in which the protection of biodiversity has a high priority (e.g., promoting public relations work for more wilderness or exemplary nature conservation in German public forests). Strategies promote biodiversity conservation through appropriate measures, which are made available to key decision-makers and policymakers [Federal Ministry for the Environment, Nature Conservation, Nuclear Safety and Consumer Protection (BMUB), 2015]. Additionally, strategies such as Article 13 of the CBD aim to promote and support understanding of the importance of biodiversity conservation and the measures required to achieve it, as well as disseminating these measures through the media and including them in educational programs [United Nations Conference on Environment and Development (UN), 1992].

In a recent European survey, respondents reported that EU initiatives should focus on restoring biodiversity and the natural environment to compensate for damages to date and better inform citizens about the importance of biodiversity (European Commission, 2019a,b). Educating people about biodiversity and the implications of biodiversity loss requires an understanding of how individuals perceive biodiversity and its associations, as well as identifying misconceptions and barriers to effective behaviors in biodiversity conservation (Bele and Chakradeo, 2021; Bernardo et al., 2021). Such an initiative would add to the existing body of knowledge in the scientific community and address various social groups’ representations, perceptions, and understandings of biodiversity and its loss. Only in the last decade has research focused on people’s perceptions of biodiversity, and their understanding of this complex concept (Bele and Chakradeo, 2021).

### 1.1. Laypeople’s representations and perceptions of biodiversity and its loss: current state of research

In the present study, the theory of social representation was applied as a theoretical framework, which was first proposed by Moscovici (1961) within social psychology (Wagner, 2020). In this theory, social representations are defined as the shared beliefs, values, attitudes, and practices of a particular social group or society (Moscovici, 2000). Shared representations help individuals understand and interpret their environment and improve communication within their social groups. Investigating social representations increases our understanding of socially relevant or problematic issues (Marková, 2008), including biodiversity and its loss, as explored in the present paper. Social representations “familiarize the unfamiliar” (Moscovici, 2000, p. 37), thereby enabling the public to make sense of scientific terms such as “biodiversity” and to align such terms with their own conceptual experience and knowledge. In a cognitive anchoring process, new information is assigned to an existing knowledge system. Over time, new ideas, experiences, and social influences can challenge and reshape existing social representations, such that social representations cannot be perceived as static or fixed (Moscovici, 2000).

The theory of social representation provides a useful framework for understanding how individuals and groups make sense of biodiversity and biodiversity loss. Several studies have investigated social representations of the concept of biodiversity with focus group discussions, open questions, photographs, or association tests, among others (e.g., Buijs et al., 2008; Levé et al., 2019; Bernardo et al., 2021; Bosone and Bertoldo, 2022). Bernardo et al. (2021) used two different methods (open questions and photographs) to investigate social representations of biodiversity in respondents from Lisbon, Portugal. The study found that a considerable number of participants were unfamiliar with the concept of biodiversity, with approximately 20% understanding biodiversity as “the diversity of animals and/or plants.” Other definitions included “related to the environment” and “nature.” Similar results were found in a study by Buijs et al. (2008), in which participants from the Netherlands, Scotland, and Germany offered rich and complex social representations in focus groups but understood biodiversity primarily in terms of species diversity and sometimes habitat diversity. Genetic diversity was rarely mentioned in participants’ shared representations. According to the study’s authors, the concepts and definitions of biodiversity among laypeople differed from those of the scientific community. Among laypeople, an understanding of biodiversity can be shaped by daily experiences, feelings, and knowledge about their environment, which together form their perceptions of biodiversity. Bosone and Bertoldo (2022) used a free-association task with French citizens to identify their common associations with biodiversity. Participants were asked to name the first three words that came to mind when hearing the term “biodiversity.” The most common responses were “nature,” “fauna,” and “flora.” Other common associations included scientific terms, science, risk, and preservation. Levé et al. (2019) explored the social representations of 1,260 French citizens by posing an open-ended question on how biodiversity should be defined. Overall, 1,065 different words were obtained. Associations named by more than 100 people mainly echoed institutional or scientific definitions such as “species,” “diversity,” and “ecosystem.”

Other studies have explored issues related to social representations, such as conceptual structures and frameworks, as well as the mental constructs of biodiversity (Turner-Erfort, 1997; Fischer and Young, 2007; Kostova and Radoynovska, 2008; Lindemann-Matthies and Bose, 2008; Dikmenli, 2010; Kilinc et al., 2013; Bakhtiari et al., 2014; Kaltenborn et al., 2016). As early as 1997, when the concept of biodiversity was still new, the study of Turner-Erfort (1997) identified a wide range of definitions for biodiversity by surveying participants in Chicago. However, only a few included elements of definitions that are currently endorsed by the scientific community. Dikmenli (2010) collected ideas about the term “biodiversity” from a group of student teachers in Turkey by using a word association test to explore their conceptual framework. The study’s results showed that the student teachers named more associations pertaining to ecosystem and species diversity and fewer associations pertaining to genetic diversity. Another study on Turkish students’ conceptions of biodiversity found that the students preferred the definition “biodiversity is the diversity of living organisms” (Kilinc et al., 2013). Among members of the Swiss public, an understanding of biodiversity as species diversity most often referred to animals and plants (Lindemann-Matthies and Bose, 2008). Kostova and Radoynovska (2008) used word associations with the prompt “biodiversity” to build conceptual structures and found various representations of biodiversity for different groups in schools, even though biology teachers taught all previously defined levels and aspects of biodiversity. Representations from the younger students focused on the emotional aspects of biodiversity (e.g., happiness, feelings, or peace of mind). Among older students, the ecological and genetic aspects of biodiversity were more widely acknowledged, while supervisors tended to name the social aspects of biodiversity such as humans, life, and social diversity (Kostova and Radoynovska, 2008). Research on perceptions of the ecological concept of forests as part of biodiversity found that individuals understood the value and role of regulation (Bakhtiari et al., 2014). The analysis of a series of group discussions with members of the public in Scotland showed that mental constructs of the concept of biodiversity increasingly included terms such as “balance,” “food chains,” and “dominance.” Additionally, the participants were aware of the irreversible loss of biodiversity (Fischer and Young, 2007). In Norway, half of the surveyed public perceived that biodiversity loss is both real and a major environmental problem (Kaltenborn et al., 2016).

In Germany, some studies have investigated social representations of biodiversity or related issues by surveying the German public. The *Nature Awareness Study 2019* found that 45% of Germans were familiar with the term “biodiversity,” had an understanding of its substantive meaning, and can named at least one of its three subcomponents [i.e., genes, diversity of species, and ecosystems; Federal Ministry for the Environment, Nature Conservation, Nuclear Safety and Consumer Protection (BMU) and Federal Agency for Nature Conservation (BfN), 2019]. The results for adolescents were similar to those in the aforementioned studies, with species diversity being the most present factor. However, in general, biodiversity was increasingly perceived as a one-dimensional concept (Schneiderhan-Opel and Bogner, 2019). Fiebelkorn and Menzel (2013) found that the prospective biology teachers in their study lacked an understanding of genetic diversity and did not view this concept as an integral component of biodiversity, and biodiversity was typically equated with species diversity. In the recent *Weleda Nature Study 2021* on biodiversity, results for Germany showed that the term “biodiversity” has a concrete meaning for many and the majority view biodiversity as worth protecting. Overall, 73% of Germans answered “yes” to the question of whether the conservation of biodiversity is important to them (Weleda, 2021). Moreover, in a survey conducted by the European Commission, German citizens indicated that pollution, man-made disasters, and climate change are the primary threats to biodiversity (European Commission, 2019a). Most respondents perceived human intervention (e.g., the destruction of forests and unlimited consumption) as a threat to biodiversity (Weleda, 2021). However, in a study of German students, the loss of biodiversity at the local level was not represented (Menzel and Bögeholz, 2009).

### 1.2. Study aim

The present study aimed to identify a socially constructed understanding of the German public regarding the concepts of biodiversity and its loss by using free word association tests and further analyzing association networks. Included in this aim is an expansion of the general knowledge of “biodiversity” by using—for the first time—the negative stimulus “biodiversity loss” with these methods. In addition to contributing to the scientific community’s understanding of the social representations and perceptions of these concepts, the results from this study may help improve campaigns for the conservation of biodiversity and enhance interdisciplinary collaboration.

Results from the aforementioned studies suggest that more associations are named when assigned to the dimensions of species and ecosystem diversity, with fewer associations for genetic diversity. Rather than create an assignment for already existing dimensions of biodiversity, the present study aimed to collect naïve associations of biodiversity and its loss. Thus, lay or nonscientific associations were not evaluated as incorrect, but as valid representations in their own right (Moloney et al., 2014), since an investigation of social representations should be focused on the way concepts such as biodiversity and its loss are understood in the public domain (Bauer and Gaskell, 2008). Existing studies nevertheless provide a good basis for interpreting and classifying our results in the context of social representations or issues related to biodiversity and its loss for the German public.

The present study used the following methodology: (1) self-reported knowledge was surveyed to gain insights into the German public’s awareness of the concept of biodiversity and its meaning; (2) free word association tests were implemented as an assessment tool since they provide a method for capturing associations by surveying social representations with a greater range of freedom than closed questionnaires. Additionally, we investigated whether there was a difference between the total number of named associations and the stimulus words “biodiversity” and “biodiversity loss” since the frequency of difference could be considered a quantitative and collective criterion in social representation theory (Lo Monaco et al., 2017). Furthermore, the categories were inductively derived from the free word associations to represent the perceptions of the German public and then generalized from the associations; (3) a novel approach in the form of an association network and its analysis (a method adapted from social network analysis) was used to visualize both the overall and the connected social representations of biodiversity and its loss, as perceived by members of the German public. Association networks can demonstrate how participants’ representations are cognitively organized and how central representations can be derived from them (Danowski, 1993). Moreover, association networks indicate which associations are frequently named together, while central representations of the networks can be identified using statistical network analysis. To the best of our knowledge, no other study to date has generated an association network that illustrates associations with biodiversity or biodiversity loss among members of the German public.

## 2. Materials and methods

### 2.1. Sample

Data collection for this study occurred in September 2021 and was conducted via an online questionnaire and the access panel of Consumerfieldwork GmbH. The panel book listed 39,306 available participants (Consumerfieldwork GmbH, 2020). Two questionnaires were available on the following topics: (1) the perception of *biodiversity*; (2) the perception of *biodiversity loss.* Quotas for age, gender, and federal state were applied to ensure a sample approaching the representativeness of the German population. The minimum age for participation was 18 years.

The surveys initially resulted in sample sizes of *N* = 177 (*biodiversity*) and *N* = 171 (*biodiversity loss*), which were subsequently processed to ensure the quality of the data. First, participants who did not correctly answer the following attention check were excluded: “*Please click ‘Strongly disagree’ on the far left to demonstrate that you are paying attention to our study.*” Additionally, the review included checking for “completeness” (Schendera, 2007) and removing data from participants who did not complete the questionnaire. Participants’ questionnaires were also checked for the “plausibility of the answer pattern.” When recurring patterns were detected in the Likert scales of the questionnaires, they were excluded from the dataset. Participants who were thought not to be serious about completing the questionnaires were removed from the sample. For this purpose, half of the median total processing time of all participants was calculated. Participants whose processing time was less than the calculated value were sorted out (Hartmann and Siegrist, 2020). Finally, participants were removed from the sample due to missing associations. Associations such as “*do not know*” were also removed from the dataset. If this filtering resulted in participants being left with no associations, they were also excluded. The total sample sizes for the final analysis were thus *n* = 131 (*biodiversity*) and *n* = 130 (*biodiversity loss*). The descriptive data of the sample are presented in Table 1.

**Table 1:** Detailed overview of the samples for the *biodiversity* (*n* = 131) and *biodiversity loss* (*n* = 130) questionnaires.

Variable	Response format	Biodiversity [%]	Biodiversity loss [%]
Gender	“Men”	44.3	43.1
	“Women”	55.7	56.9
Age	“Open question” 18–20 years	0.8	1.5
	21–24 years	5.4	5.4
	25–39 years	26.1	29.3
	40–59 years	37.4	35.2
	60–64 years	8.3	5.5
	> 65 years	22.4	23.0
Education level	“Secondary education”	26.7	20.7
	“Intermediate school certificate”	31.3	29.8
	“Advanced technical college entrance qualification”	6.9	11.5
	“General qualification for university entrance”	28.2	37.7
	“Another school degree”	3.8	0.8

Sociodemographic data were collected according to the specifications of the Federal Statistical Office [Destatis (Federal Statistical Office), 2021].

The sample of *biodiversity* was composed of 58 men (44.3%) and 73 women (55.7%). The sample of *biodiversity loss* was composed of 56 men (43.1%) and 74 women (56.9%). Compared to the gender distribution in Germany [49.3% men and 50.6% women; Destatis (Federal Statistical Office), 2021], it is noticeable that the proportions of males in the samples were slightly smaller than those of females. The average ages of the participants were 49.2 years (*SD* = 16.8; *biodiversity*) and 47.8 years (*SD* = 17.0; *biodiversity loss*). Compared to the German population [44.7 years, Destatis (Federal Statistical Office), 2022] the average ages of the samples were significantly higher. This discrepancy could be explained by setting the minimum age of the participants at 18 years. When considering the level of education, measured by the highest school-leaving qualification (‘General qualification for university entrance’), the level of education in the samples was lower (*biodiversity:* 28.2%) or higher (*biodiversity loss:* 37.7%) than the average highest level of education of the German population [33.5%; Destatis (Federal Statistical Office), 2020].

### 2.2. Questionnaire

The questionnaires were created using the SoSci Survey (v. 3.2.30) online platform (Leiner, 2019) and included the following sections:

(1) *Self-reported knowledge*: Following the *Nature Awareness Study 2019* published by the Federal Ministry for the Environment, Nature Conservation, Nuclear Safety and Consumer Protection (BMU) and Federal Agency for Nature Conservation (BfN), 2019, participants were asked whether the term “biodiversity” was previously known (*“Were you familiar with the term ‘biodiversity’ before the association test?”*).
(2) *Free word association test*: Within the *biodiversity* questionnaire, participants were asked for free word associations for the stimulus word “biodiversity.” Within the *biodiversity loss* questionnaire, participants were asked for the stimulus word “biodiversity loss.” Notably, the term “biological diversity” was always used instead of biodiversity [in German: *Biologische Vielfalt* instead of *Biodiversität*] in this study. By changing the original Latin and educational language term, we expected it to be easier for participants to name associations. Finally, participants were given one working definition of biodiversity and its loss to allow for equal prerequisites of the participants for the processing of further questions.
(3) At the end of the questionnaire, *sociodemographic data* were collected from the participants. These data included the gender, age, postal code of the current place of residence, information on school-leaving qualifications, monthly net income, political party one would currently vote for, place of origin, and current place of residence. The questionnaire contained additional scales that are irrelevant to the presentation of the data in this publication.

#### 2.2.1. Free word association test

Free word association tests are a viable method for determining participants’ attitudes and perceptions about an object (Szalay and Deese, 1978). Lo Monaco et al. (2017) described word association tasks as one of the main methods for collecting the content of social representations (Lo Monaco et al., 2017, p. 309). Wagner et al. (1996, p. 334) further described the technique as having unrestricted access to mental representations. Moreover, word association tests reveal scientific conceptual structures that depend on scientific education on the one hand and social environment on the other (Kostova and Radoynovska, 2008). In general, an association could be concrete or abstract, and it could be expressed in many ways (e.g., by a verb, a noun, or an adjective; Kahnemann, 2012).

In the present study, we investigated possible distinctions between the associations of the two association tests for the stimulus words *biodiversity* and *biodiversity loss.* The association tests of both questionnaires started with an introductory text informing the participants about the anonymity of the test. Participants were then asked to express their associations freely and spontaneously. Additionally, the associations were to be written down in the order in which they came to the participants’ minds. Participants were told to avoid chain responses, loose phrases, and complete sentences. Next, the following prompt was shown: “*When you are ready for the association test, click ‘Next.’ The test will then begin immediately.*” Once participants clicked “Next,” the processing time of 1 minute began, and 10 response boxes appeared. As soon as the participants submitted an answer, another answer field was added. Thus, there were always 10 answer fields available. The following text was also displayed above the answer fields: “*What associations come to mind when you hear the term ‘biodiversity’/‘biodiversity loss’?*” “*Please write down here any terms that come to mind. Please write only one word per line.*”

The remaining time was displayed with the help of a countdown timer above the question sheet page. According to Szalay and Deese (1978), it is useful for an association test to introduce a time limit for the submission of the associations. The associations given are influenced if participants are not under any time pressure (Siipola et al., 1955). Therefore, it is recommended that participants should be given 1 minute to state associations (Szalay and Deese, 1978).

Notably, the association tests for both questionnaires were placed at the very beginning to avoid possible influences from other questions. All associations were translated from German into English by employing the DeepL Pro translation service and several biological dictionaries, (e.g., a dictionary for animal names; Cole, 2008, 2015a,b).

### 2.3. Data analysis

#### 2.3.1. Coding

First, the associations were checked for spelling and grammatical errors. The terms were converted to the singular for standardization. Except for a few associations, such as those that describe a variety by their form in the plural, remained in the plural (e.g., “species,” “races”). In this manner, it was possible to combine terms that were singular as well as plural into one code. All rules that were applied to edit the associations can be requested from the first author. The adjustments were made in Microsoft Excel. Responses from participants that contained two associations were included as two single-word associations. Subsequently, the Excel files were imported into MAXQDA (v. 20.4.0) (VERBI Software, 2021). Here, identical associations were combined into one code. In this manner, it was possible to locate the frequency of individual associations. This was followed by inductive category formation. Deriving categories inductively involves starting with raw data and then systematically organizing it into meaningful categories, often through a process of coding or categorization. This may involve identifying common themes or patterns across different responses and looking for recurring words or phrases (Rädiker and Kuckartz, 2019). Based on the available associations of both questionnaires, 26 categories could be assigned to them. For example, the associations “animal,” “insect,” and “dog” were coded into the category “animals.” For the category “plants,” associations such as “plant,” “flower,” and “tree” were coded. Furthermore, the associations “bee extinction,” “insect extinction,” or “forest decline” were coded into the category “animal and plant extinction.” Associations, that could not be clearly interpreted were sorted into the category “other” (e.g., “freedom,” or “time”). The complete codebook is available in the Supplementary material.

Subsequently, the intercoder-reliability was checked in MAXQDA (v. 20.4.0). For this purpose, an independent person recoded the associations into the existing categories based on the associated definitions. The two independently coded document groups were merged in MAXQDA and then compared using the intercoder match function. The goal was to achieve a match of at least 80%. Additionally, *kappa* according to Brennan and Prediger (1981) was calculated in MAXQDA. This characteristic value considers the likelihood of agreement among the coders (Rädiker and Kuckartz, 2019). The Brennan and Prediger (1981) *kappa* value can range from −1 to 1, with 0% agreement among coders having a value of −1. When *kappa* takes the value of 1, the agreement among coders is 100%. A value of 0 represents chance (Brennan and Prediger, 1981). A *kappa* value from 0.61 to 0.80 is considered “substantial,” or good. When *kappa* has a value from 0.81 to 1, it is considered “almost perfect” (Landis and Koch, 1977). To improve the agreement, non-matches were discussed again, with coders agreeing on a category. Only associations that were mentioned at least three times were considered. Conducted intercoder–reliability testing in MAXQDA revealed the following: The resulting *kappa* value for *biodiversity* was “almost perfect” (κ = 0.82). The resulting *kappa* value for *biodiversity loss* was “substantial” (κ = 0.72). After discussion of doubtful cases between the coders and subsequent adjustment, the intercoder–reliability revealed higher kappa values (κ = 0.87; almost perfect; *biodiversity* / κ = 0.79; substantial; *biodiversity loss*). The protocol for discussing the nonconformity of associations can be requested from the first author.

#### 2.3.2. Association network

Following the social network analysis, in this study, networks were used to visually replicate the associations of participants and reveal representations for the stimulus words *biodiversity* and its *loss*. In addition, networks demonstrate how participants’ ideas are cognitively organized, and central concepts could be identified (Danowski, 1993). For psychologists, ideas are nodes in a vast network called associative memory, in which each idea is connected to many other ideas (Kahnemann, 2012). To investigate which associations frequently occur together in the mental concepts of participants, association networks were created in Gephi (v. 0.9.7; Bastian et al., 2009), following the procedure used by Vlasák-Drücker et al. (2022). From the free word association tests, the code–relations–browser was first generated in MAXQDA for each, whereby only those associations that were mentioned at least five times were considered. The threshold value was selected to ensure improved network clarity. The code–relations–browser matrices were then imported into Gephi (v. 0.9.7). To create the network, several layouts were available that determined the order of the associations. Here, the “Fruchterman Reingold” option was used. For the sake of clarity, the edges (i.e., connections between the associations) were filtered according to weight. Thus, associations that were named with each other three times (*biodiversity*) or two times (*biodiversity loss*) do not share visible connecting lines in the graphic. This reduced number of edges resulted in better visualization of the network. Furthermore, the networks were edited in Inkscape v. 1.2.1 (2022). Nodes and edges were colored, and the words corresponding to the four largest categories and further categories (“other”) were colored in the networks.

#### 2.3.3. Statistical tests

Statistical data analysis was conducted using SPSS^®^ software (IBM^®^ v. 27). To investigate whether there is a significant difference between the total number of mentions in the two questionnaires, their normal distribution was first calculated. After it was determined that the data were not normally distributed, the Mann–Whitney U-test was performed.

In addition, analysis was conducted for statistical network measures using Gephi (v. 0.9.7). The assessment was based on key approaches, that helped understand how a network is structured: *graph density*; *degree centrality*; *betweenness centrality*. First, *graph density* measured the level of connected edges (in our case, the connection lines between associations) within a network relative to a total possible value. Graphs with values near 1 are considered dense, while graphs with values near 0 tended to be sparse graphs (Cherven, 2015; Borgatti et al., 2022). Moreover, centrality statistics were investigated. They provided the framework to compare the roles played by various nodes (in our case, associations) within a single network. *Degree centrality* was investigated for undirected graphs, which provided importance for the number of direct connections (degrees) one node had to other nodes’ influence within the network. It was useful to identify strongly connected associations (Cherven, 2015; Borgatti et al., 2022). *Betweenness centrality* showed which nodes functioned as a “bridge” between the nodes of a network. All of the shortest paths were identified and summarized based on how often a node passed the shortest path. The identification of *betweenness centrality* was useful to predict the flow of the network based on associations (Cherven, 2015; Borgatti et al., 2022). All centrality measures were normalized to simplify the comparison.

## 3. Results

### 3.1. Self-reported knowledge of biodiversity

**Figure 1:**
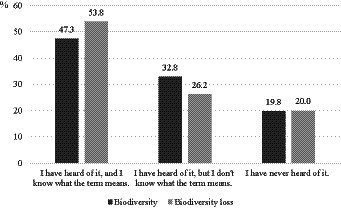
Self-reported knowledge of participants from questionnaires on *biodiversity* (*n* = 131) and *biodiversity loss* (*n* = 130) for the term “biodiversity.” Question asked following the *Nature Awareness Study 2019* published by the Federal Ministry for the Environment, Nature Conservation, Nuclear Safety and Consumer Protection (BMU) and Federal Agency for Nature Conservation (BfN) (2019). Frequencies are presented as percentages.

For surveying self-reported knowledge, participants were asked whether they were familiar with the term “biodiversity” and knew what it meant (Figure 1). The results showed, that in total, 47.3% (*biodiversity*, *n* = 62) and 53.8% (*biodiversity loss, n* = 70) of the participants indicated that they had already heard of biodiversity and knew what it meant. Moreover, 32.8% (*biodiversity*, *n* = 43) and 26.2% (*biodiversity loss*, *n* = 34) of participants indicated that they had already heard of the term “biodiversity” but had no prior knowledge of it. The remaining 19.8% (*biodiversity loss*, *n* = 26) and 20.0% (*biodiversity loss*, *n* = 26) of participants were completely unfamiliar with the term “biodiversity.”

In addition, a Mann–Whitney U-Test was performed to determine whether a statistical difference was observed between the two surveyed groups in terms of self-reported knowledge. No statistically significant difference was observed in the self-reported knowledge of *biodiversity* and *biodiversity loss* (*U =* 8098.0, *Z* = −0.64, *p* = 0.53) between the two surveyed groups. Thus, the self-reported knowledge baseline for the following analysis was the same for both groups.

### 3.2. Social representations of Germans regarding biodiversity and its loss

The free word association test was used to survey social representations. The total number of associations named by participants was 1,222. Of these, 652 distinct associations with *biodiversity* and 570 distinct associations with *biodiversity loss* remained. A list of all associations is available in the Supplementary material. On average, 5.02 (*SD* = 2.55) associations were named per participant for *biodiversity*, while 4.3 (*SD* = 2.31) associations were named for *biodiversity loss*. A maximum of 12 named associations (*biodiversity*) or 11 named associations (*biodiversity loss*) was achieved in the given time of 1 minute by a small number of participants.

The 10 most frequently mentioned associations of the two questionnaires as well as their relative frequencies are presented in Table 2. For *biodiversity* these account for 32.7% of the total associations (*n* = 213). For *biodiversity loss*, these account for 24.7% (*n* = 141). The three most frequently mentioned associations for biodiversity were “animal” (*n* = 50), “plant” (*n* = 39), and “nature” (*n* = 26). For *biodiversity loss*, the associations were “species extinction” (*n* = 38), “climate change” (*n* = 16), and “plant” (*n* = 15). A Mann–Whitney U-Test was calculated to determine whether there were differences in the total number of mentioned associations between participants in the two questionnaires for *biodiversity* (*M*_Rank_ = 140.94) and *biodiversity loss* (*M*_Rank_ = 120.98). There was a statistically significant difference in the total number of mentioned associations between *biodivers*ity and *biodiversity loss* (*U* = 7213.0, *Z* = −2.16, *p* = 0.03), with a small effect (*r* = 0.13; Cohen, 1988).

**Table 2:** Top 10 associations of *biodiversity* (*n* = 652) and *biodiversity loss* (*n* = 570) with absolute and relative frequency.

Biodiversity			Biodiversity loss		
Association	Absolute frequency	Relative frequency	Association	Absolute frequency	Relative frequency
Animal	50	7.7	Species loss	38	6.7
Plant	39	6.0	Climate change	16	2.8
Nature	26	4.0	Plant	15	2.6
Human	18	2.8	Insect	13	2.3
Flower	16	2.5	Bee	12	2.1
Species diversity	15	2.3	Animal	11	1.9
Environment	14	2.1	Extinct	10	1.8
Insect	13	2.0	Nature	9	1.6
Species	12	1.8	Monoculture	9	1.6
Bird	10	1.5	Human	8	1.4

Relative frequency is presented as a percentage.

### 3.3. Categorized social representations of biodiversity and its loss

To answer, which categories can be derived from the overall named associations to the prompts *biodiversity* and *biodiversity loss*, the 10 categories to which the most associations were assigned are shown for both questionnaires (Table 3), including the absolute and relative frequencies. The three categories with the most commonly named associations for *biodiversity* were “animals” (*n* = 108), “plants” (*n* = 79), and “diversity” (*n* = 75). For *biodiversity loss*, these categories were “other” (*n* = 82), “anthropogenic causes” (*n* = 69), and “animals” (*n* = 65).

**Table 3:** Top 10 categories of *biodiversity* and *biodiversity loss* with absolute and relative frequency.

Biodiversity			Biodiversity loss		
Category	Absolute frequency	Relative frequency	Category	Absolute frequency	Relative frequency
Animal	108	16.6	Other	82	14.4
Plants	79	12.1	Anthropogenic causes	69	12.1
Diversity	75	11.5	Animal	65	11.4
Habitat	54	8.3	Species loss	62	10.9
Other	51	7.8	Animal and plant extinction	58	10.1
Food	42	6.4	States	27	4.7
Conservation	37	5.7	Plants	21	3.7
Nature	34	5.2	Environment	18	3.2
Human	28	4.3	Habitat	17	3.0
States	24	3.7	Diversity	17	3.0

The total number of categories is 26. The full list of the created categories with short definitions and complete definitions can be found in the Supplementary material. Relative frequency is presented as a percentage.

### 3.4. Association networks

**Figure 2:**
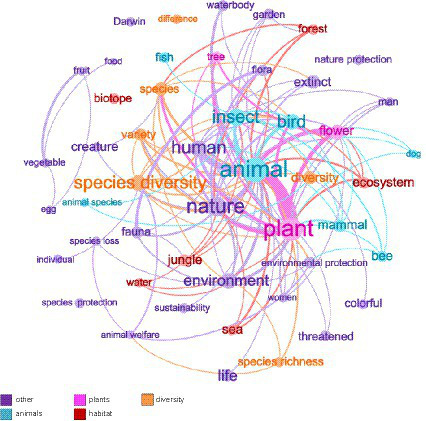
Association network for *biodiversity*. Only associations named five or more times were included in the network. The more frequently an association was named, the larger it appears in the network. The more often an association is named with another, the thicker the edge between them. For the sake of clarity, the edges (i.e., connections between associations) were filtered according to weight. Thus, associations that were named with each other fewer than two times do not share visible connection lines in this graphic. Furthermore, the four categories that comprise the most associations have been colored, along with the coloring of all other categories (i.e., “other”). Associations such as “Darwin,” “difference,” “biotope,” “species conservation,” and “nature conservation” were mentioned five times, but less than two times with another association. Accordingly, they stand alone in the network.

**Figure 3:**
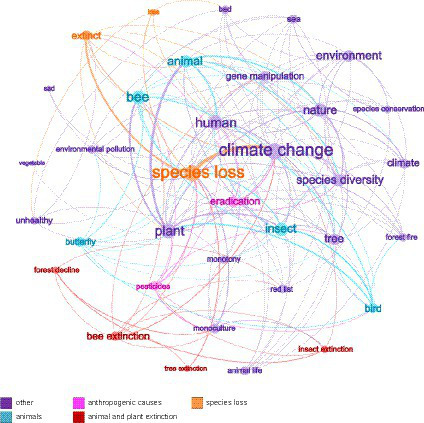
Association network for *biodiversity loss.* Only associations named five or more times were included in the network. The more frequently an association was named, the larger it appears in the network. The more often an association is named with another, the thicker the edge between them. For the sake of clarity, the edges (i.e., connections between associations) were filtered according to weight. Thus, associations that were named with each other fewer than three times do not share visible connection lines in this graphic. Furthermore, the four categories that comprise the most associations have been colored, along with the further coloring of all other categories (i.e., “other”).

The results of association networks visualized the overall and connected social representations of *biodiversity* and *biodiversity loss* in the German public. Associations that were frequently named together in the questionnaires are shown in Table 4. The associations “plant” and “animal” were most frequently mentioned together for *biodiversity* (*n *= 25). For *biodiversity loss*, the associations “species extinction” and “climate change” occurred most frequently together (*n* = 11; Figures 2, 3). Additionally, the associations were colored based on their membership in the previously created categories. Only the four largest categories were considered, plus “other” categories. In the association network for *biodiversity*, it is noticeable that associations from the categories “animals” and “plants” frequently occurred together. Associations from the category “food” were also frequently mentioned together. For *biodiversity loss*, associations from the categories “anthropogenic causes” and “species extinction” frequently occurred together. Also, associations from the category “animals” were often named together.

**Table 4:** Top 10 most frequently named association word pairs for *biodiversity* and *biodiversity loss.*

Biodiversity		Biodiversity loss	
Word pair	Frequency	Word pair	Frequency
Plant – Animal	25	Species loss – Climate change	11
Human − Animal	13	Plant – Animal	7
Nature − Animal	11	Extinct – Species loss	5
Nature − Plant	9	Plant – Bee	5
Flower − Animal	8	Plant – Insect	4
Bird − Animal	7	Plant – Bird	4
Human − Plant	7	Nature – Animal	4
Bird − Plant	6	Human – Animal	3
Environment − Plant	6	Monoculture– Species loss	3
Environment − Animal	6	Insect – Species loss	3

Results for the statistical network analysis of the association networks are provided in Table 5. The *graph density* of the two association networks shows that 28.8% (*biodiversity*) and 29.4% (*biodiversity loss*) of the nodes are connected. It can be assumed that this network is rather sparse since the value for *graph density* tended toward 0 (both approximately 0.30) instead of 1. For *degree centrality,* there is a strong differentiation between the nodes for both *biodiversity* and *biodiversity loss*, as seen from the variance of the degree values (*biodiversity*: 0.08 to 0.92; *biodiversity loss*: 0.03 to 0.62). Additionally, for *betweenness centrality*, significant variation was observed in the centrality values. Both networks seem to require “bridge” nodes. For *biodiversity*, the nodes are “animal” and “plant” (*betweenness* = 0.28; 0.16), while for *biodiversity loss*, these are “species loss” and “climate change” (*betweenness* = 0.17; 0.12). These associations are also centrally located in the networks. Associations such as “animal welfare” (*biodiversity*) and “forest decline” (*biodiversity loss*) have a low *degree* and *betweenness centrality*. These associations are in the periphery of the network (Figures 2, 3).

**Table 5:** Statistical network analysis for association networks of *biodiversity* (nodes = 51, edges = 367) and *biodiversity loss* (nodes = 35, edges = 175).

Biodiversity			Biodiversity loss		
Graph density	0.288		Graph density	0.294	
	Degree	Betweenness		Degree	Betweenness
Animal	0.92	0.28	Species loss	0.62	0.17
Plant	0.84	0.16	Climate change	0.62	0.12
Nature	0.66	0.05	Plant	0.53	0.08
Insect	0.56	0.04	Human	0.53	0.06
Human	0.52	0.02	Insect	0.47	0.04
Species diversity	0.48	0.02	Bee	0.47	0.03
[…]			[…]		
Individual	0.14	0.0003	Insect extinction	0.15	0.0077
Animal species	0.14	0.0009	Loss	0.15	0.0053
Animal welfare	0.12	0.0001	Forest decline	0.12	0.0009
Dog	0.12	0.0002	Sad	0.06	0.0006
Egg	0.10	0.0018	Tree extinction	0.06	0
Food	0.08	0.0004	Vegetable	0.03	0

All centrality measures were normalized. For better comparability, the presets of edge weight for the calculation of the statistics have been omitted. The presets are only used for the clarity of presenting the networks in Figures 2, 3. Original Gephi networks without any preset edge weight and the total results for statistical network analysis are provided in the Supplementary material.

## 4. Discussion

Our study’s objective was to determine how the German public perceives biodiversity and its loss. We accomplished this by conducting free word association tests and analyzing association networks. By using “biodiversity” and—for the first time—the negative stimulus “biodiversity loss” our results should contribute to the scientific community’s understanding of the social representations and perceptions of these concepts. The findings of this study could be used to enhance biodiversity conservation campaigns and promote collaboration across different fields of study.

### 4.1. Self-reported knowledge of biodiversity

The results of participants’ self-reported knowledge showed similar findings as the 2019 German *Nature Awareness Study* [Federal Ministry for the Environment, Nature Conservation, Nuclear Safety and Consumer Protection (BMU) and Federal Agency for Nature Conservation (BfN), 2019] and the 2019 Eurobarometer (European Commission, 2019a). In our study, approximately half of the participants believed they knew the term and understood its content. This result differs from slightly fewer participants in Germany (45%) and Europe (41%) who reported knowing and understanding the term. While these two studies reported an increase in awareness of the term, they also noted that more than half of the respondents did not yet know or had never heard of the term’s meaning. Nevertheless, the participants in our study who did not know the term and its meaning were able to name associations with biodiversity and its loss.

### 4.2. Social representations and networks

Social representations were surveyed using free word associations. The resulting presentations in association networks revealed the German public’s broad understanding of biodiversity and its loss. Our analysis suggests that individuals possessed certain notions about biodiversity and its loss, which could be assessed as either positive, negative, social, everyday life, or scientific and technical. Naïve associations suggested that biodiversity appears as a comprehensive and multidimensional phenomenon that could evoke varied responses in different people depending on where they come from and the ecosystems with which they are closely associated (Zemits, 2006).

However, a detailed look at our results revealed various conceptualizations that may depend on individual and cultural backgrounds and the shared knowledge of a group (Fiebelkorn and Menzel, 2013). Further investigations could differentiate shared social representations within our surveyed group of German publics according to age, education level, or political preference. Notably, social representations are regarded as static to a limited extent and the results of the present study may only apply in the short term (Moscovici, 2000). The associations of biodiversity and its loss were aggregated and represented across the entire sample from the German public in the association networks. We assumed that many of the associations and their connections could be elements of social representations of biodiversity and its loss among the German public, in this or a similar way. Furthermore, the association networks are not simply a common representation of “word clouds.” In the present study, they showed the connections of all associations in a network and, more importantly, how closely they are linked to each other.

#### 4.2.1. Animals and plants: social representations of *biodiversity*

The word stimulus exercise on *biodiversity* showed that animals and plants were the most frequently mentioned associations in the social representations of the participants. Also, network statistics indicated that these two associations (“animal” and “plant”) had the most connections to other associations and were identified as bridges from which further associations emanated, thereby predicting the flow in the network. In line with our results, Lindemann-Matthies and Bose (2008) reported that biodiversity was most frequently defined with the terms “animal” and “plant.” In our study, participants additionally reported many specific taxa, such as birds and insects, and somewhat fewer mammals. In contrast, plant taxa such as mosses, ferns, or flowering plants were less commonly provided and were thus not visible in the network or among the top 10 associations. In the association network, only one association from “plant” to “flower” was found. Interestingly, plants were at the center of the association network, alongside animals, even though many people tend to overlook the importance of plants in the biosphere—a phenomenon known as “plant blindness” (Jose et al., 2019).

Although certain mammals, including so-called “charismatic megafauna,” are often used as flagship species for biodiversity protection (Albert et al., 2018; McGowan et al., 2020), insects and birds were more frequently associated with biodiversity than mammals in the present study. This could be due to the attention currently paid to these groups of organisms by the promotional campaigns of the largest German NGO, the Nature and Biodiversity Conservation Union [Nature and Biodiversity Conservation Union (NABU), 2023]. For example, the NGO’s logo depicts a stork, with groups of birds and insects being displayed in their ongoing campaigns (e.g., the election of the bird/insect of the year). Moreover, the most popular flagship species, such as tigers, lions, and elephants (Albert et al., 2018), are not native to Germany, and thus possibly had little presence among the laypeople surveyed. Leandro and Jay-Robert (2019) focused on insects as featured animals of diversity and found that mammals were more entrenched in young adults’ concepts of biodiversity. However, this was not demonstrated by the results of the current study.

The decline in bird and insect species in Germany is concerning [International Union for Conservation of Nature (IUCN), 2022]. However, recent research suggests that the German population’s attitude and willingness to protect these species is increasingly positive (Dörge et al., 2022; Eylering et al., 2022). Featuring birds and insects in educational campaigns on biodiversity conservation and strengthening links to the concept of biodiversity could thus be beneficial. A public campaign highlighting birds and insects could strengthen other related associations suggested by the network. After starting with birds and insects, efforts could be geared toward “plants” such as helping to reduce the aforementioned plant blindness or focusing on insect “species diversity” to increase the visibility of this particularly species-rich but often endangered group.

Notably, participants seemed to perceive only macroscopically visible organisms as part of biodiversity. Microorganisms such as fungi and protists were not at all integrated with people’s associations despite making up an important component of biodiversity, with losses of these organisms having been recorded [International Union for Conservation of Nature (IUCN), 2022].

Overall, diversity terms such as “species diversity,” “variety,” or “species richness” were socially associated with biodiversity, similar to several other studies (Buijs et al., 2008; Dikmenli, 2010; Kilinc et al., 2013; Schneiderhan-Opel and Bogner, 2019). Furthermore, 93% of German participants in the *Nature Awareness Study* thought they knew the meaning of biodiversity and associated biodiversity with animal and species diversity [Federal Ministry for the Environment, Nature Conservation, Nuclear Safety and Consumer Protection (BMU) and Federal Agency for Nature Conservation (BfN), 2019]. It appears that these participants replaced the term “biodiversity” with a closely related word or synonym. The data suggested that the term “biodiversity” was most often interpreted as a synonym for “species diversity,” as described by Menzel and Bögeholz (2009).

Social representations related to the genetic dimension were limited in number. In the top 10 associations and *biodiversity* networks, no associations could be directly attributed to the scientifically defined genetic dimension. In the *biodiversity loss* network, only “gene manipulation” was apparent. Other studies have shown that the concept of genetic diversity was less pronounced (Buijs et al., 2008; Dikmenli, 2010; Fiebelkorn and Menzel, 2013). In contrast, a study by Kostova and Radoynovska (2008) found that teachers, and especially older tenth-grade students, named associations for genetics—a finding that is likely due to the school curricula, which could include genetic diversity as a sub-dimension of biodiversity. The interviewed group can also be assigned to the social context of the school. Our sample did not directly interview students; thus, their social representations from the genetic field may be underrepresented.

Interestingly, the results of the association network for *biodiversity* showed that associations for the category “food” were often named together, particularly since the participants built their networks without any connection to the main network. Presumably, these food associations were named chain responses in which the participants did not return to the initial term in the free word association test.

Overall, social representations of biodiversity in the German public appear to be multidimensional and multifaceted, even if they do not cover all the facets included in the scientific definitions. Most associations were not related to the loss or threat of biodiversity, which underlines the importance of a second stimulus that focuses more directly on loss. In summary, social researchers and policy makers can be confident that the general public has a broad understanding of biodiversity (Levé et al., 2019), which is a good starting point for individuals to recognize the consequences of biodiversity decline and could increase their willingness to support biodiversity conservation efforts and adopt sustainable practices.

#### 4.2.2. Species loss and climate change: social representations of *biodiversity loss*

The word stimulus exercise on *biodiversity loss* showed that “species loss” and “climate change” were the most frequently mentioned associations in the social representations of the participants. Additionally, network statistics indicated that these two associations (“species loss” and “climate change”) had the most connections to other associations and were identified as bridges from which further associations emanated, thereby predicting the flow of the network. This observation suggests that—climate change—one of the key drivers of biodiversity loss caused by human behavior—was present in the participants’ thought processes and social representations of biodiversity loss (Mohaupt-Jahr and Küchler-Krischun, 2008; European Commission, 2019a). Hence, a competing environmental problem was at the center of the network and was likely used as a substitute association in this case. Although climate change and biodiversity loss are two related but different issues (Bosone and Bertoldo, 2022), the concept of climate change could be far more present in the public’s perception than biodiversity loss, with the latter potentially being perceived as a lesser global environmental problem than climate change. These related issues may be difficult to separate because biodiversity loss could be perceived as just one aspect of other environmental problems (Kaltenborn et al., 2016; Legagneux et al., 2018). The participants in the study may have had difficulties in distinguishing between the related environmental issues of biodiversity and climate change. The perception of a close relationship between these two environmental issues was also shown in the recent *Weleda Nature-Study 2021* (Weleda, 2021), in which 53% of participants believed that intact biodiversity plays an important role in slowing down climate change. Conversely, even more participants (82%) were convinced that the loss of biodiversity accelerates climate change.

Therefore, as suggested by some researchers, it seems logical to embed the communication of biodiversity loss within the framework of the climate crisis (Veríssimo et al., 2014). However, due to the many other causes of biodiversity loss, we believe it is more appropriate to give the biodiversity crisis the same emphasis as that of climate change in politics, public discourse, and the media. Legagneux et al. (2018) noted that the inherent bias in communications about climate change and biodiversity is largely due to the Intergovernmental Panel on Climate Change (IPCC) being introduced twenty years before the Intergovernmental Platform on Biodiversity and Ecosystem Services (IPBES). The amount of news and information about climate change in the media has increased over time. This trend suggests that coverage and attention toward issues of biodiversity may also increase (Legagneux et al., 2018). To raise awareness and drive action on biodiversity loss, communication strategies that have been successful in addressing climate change could be employed. For example, these may include connecting people’s values, using compelling narratives or stories, and disseminating effective imagery about threatened species to communicate action on biodiversity as a form of social belonging (Sippel et al., 2022).

Interestingly, with regard to *biodiversity loss*, the network showed that the terms “species loss – climate change – human” were very central to the network. A possible explanation for this observation is that the study participants considered climate change to be human-caused and that a perception of species loss was consequently formed. A report by the International Union for Conservation of Nature (IUCN) describes how species are already being affected by anthropogenic climate change, the rapid onset of which is limiting the ability of many species to adapt to their environments [International Union for Conservation of Nature (IUCN), 2019]. Climate change currently affects at least 10,967 species on the IUCN Red List of Threatened Species, thus increasing the likelihood of their extinction [International Union for Conservation of Nature (IUCN), 2019]. For biodiversity conservationists, the findings that humans may also perceive themselves as “polluters” in the concept of biodiversity could be an important factor to consider in communications about biodiversity. However, it must simultaneously be assumed that even if people do not believe that climate change is human-caused, species extinction is also associated with climate change.

Although changes in land use are one of the paramount causes of biodiversity loss (Sala et al., 2000; IPBES, 2019), social representations of *biodiversity loss* showed little association with this factor. This finding contradicts the assumption that, over the past few centuries, changes in land use have had a far greater impact on ecological variables than climate change. Although land use seems not to be directly related to climate change, the effects of climate change have forced inhabitants in some regions to alter land use practices that ultimately affect ecosystems (Dale, 1997). Similarly, invasive alien species severely impact biodiversity (IPBES, 2019). Neither land-use changes nor invasive alien species were present in the social representations of the participants, even though these factors are assumed to directly result in biodiversity loss. Lipták et al. (2023) showed that the majority of the Czech and Slovak populations recognize that such invasions are a threat to native biodiversity. The authors suggested that access to self-education, particularly regarding invasive and protection measures, should be facilitated. Smartphone applications that provide comprehensive information on biological invasions and species identification guidance could be beneficial for users (Verbrugge et al., 2021). Moreover, the perception of the threat to biodiversity posed by invasive species may occur at the regional level (Lipták et al., 2023). In this case, further study of social representations of people in different regions would be required.

The fact that insects and bees appeared in the perceptions of participants for the term *biodiversity loss* is striking. This perception may be due to the sharp decline in insect populations in recent decades (Hallmann et al., 2017; Seibold et al., 2019). A study that used the stimulus word “insects” in a free word association test, suggested that the public seemed to be aware of the benefits of insects (Vlasák-Drücker et al., 2022). Moreover, respondents mainly associated “bees” with insects. Another study found highly positive attitudes toward the conservation of bees, and the authors recommended using bees as a flagship species to promote the local conservation of pollinating insects and to conserve biodiversity (Schlegel et al., 2015; Schönfelder and Bogner, 2017). These results suggest a need to highlight participants’ representations of insects—particularly bees—for conservation communications that focus on the prevention of biodiversity loss. However, since participants’ understanding of biodiversity loss may be constantly changing, it is also important to investigate social representations of the context of biodiversity and its loss on an ongoing basis.

#### 4.2.3. Comparison of social representations of *biodiversity* and *biodiversity loss*

Commonalities can be found in both prompts in the top 10 associations, in the top 10 categories formed, and in the word pairs. This suggests overlaps that may be helpful in biodiversity conservation campaigns by indicating what is already most associated with biodiversity and its loss.

But results for the stimulus terms *biodiversity* and *biodiversity loss* revealed also many differences. First, a difference was observed in the associations between *biodiversity* and *biodiversity loss*. Significantly fewer associations were named for *biodiversity loss*. A possible reason for this finding is that the term “biodiversity” may cause difficulties for respondents; thus, naming associations for its loss would be difficult. Nevertheless, it should be emphasized that an association test on *biodiversity loss* was a unique feature of this study, and many associations were made.

Second, general differences in associations between *biodiversity* and *biodiversity loss* can be found in the connotations of the two terms’ associations. Compared to the term *biodiversity*, negative associations such as “species loss” and “climate change” were mostly found with the term *biodiversity loss*. The biggest categories for *biodiversity loss* also contained associations describing human causes for the loss of biodiversity, along with associations addressing the extinction of species. More positive associations were mentioned for *biodiversity*, including “species protection,” “species richness,” and “sustainability.” In general, fundamental differences appeared to exist in the perception and understanding of biodiversity and its loss. The associations had a range close to the dimensions of working definitions such as “species diversity” to different habitats such as “jungle,” or “sea” to social representations such as “skin aging,” “love,” and “food.”

Third, association networks clearly differed in terms of visualization. The *biodiversity* association network seemed much more densely populated, although its density differed only slightly from the values in *biodiversity loss* association network. However, more associations in the *biodiversity* network—and thus more connections—had stronger connections between individual associations. For example, “plant” and “nature” had high degrees of centrality in that these associations were frequently mentioned together. For *biodiversity loss*, which also contained “plant,” and “nature,” the associations were much weaker. The *biodiversity loss* network also showed many—though weaker—connections between individual associations. In both networks, terms with high betweenness centrality (as described above) were found in the center, just as lower betweenness centrality was found in the periphery, which is a common phenomenon (Cherven, 2015).

A particularly common feature of both networks was the association “human,” which was anchored in the center of both networks and displayed a high degree and betweenness centrality. Presumably, humans are viewed as a factor in biodiversity and are understood to be an integral feature of the biodiversity concept, whether positive or negative. However, the respondents possibly perceived humans as the primary cause of biodiversity loss. When interpreting the present results, additional research on social representations may be required to investigate the role of humans in relation to their contact with nature. According to Bosone and Bertoldo (2022), individuals who frequently engage with nature had a greater awareness of the threat to biodiversity posed by human activities. Furthermore, such individuals tend to perceive this threat as being more imminent when compared to those who have limited exposure to nature. Also, people’s own experiences of nature, and the feelings and impacts associated with it, could broaden their understanding of biodiversity and its loss (Levé et al., 2019).

Fourth, in the survey of associations on biodiversity loss, not only the stimulus word had a negative connotation, but the title of the questionnaire also framed the loss of biodiversity. A recent study, in the environmental field showed that negative framing can attract the attention of individuals in the general population; for example, through the image-framing of human-caused impacts on the environment (Salazar et al., 2022). For communicating results on biodiversity and its loss via framed messages, Kusmanoff et al. (2020) suggested that messages must include an emphasis on things that matter to the audience (e.g., by using “bridge” associations from the participants), reduce the psychological distance (e.g., providing temporal or spatial examples where biodiversity loss is obvious), exploit useful biases (e.g., between associations of technical or social dimensions), and, where possible, test different messages that communicate biodiversity and its loss. Nevertheless, it was noticeable that aspects of protection were more often mentioned for *biodiversity*, (e.g., “species protection,” “nature protection,” and “animal welfare”). However, this does not mean that naming a corresponding association alone influences environmentally protective behavior.

### 4.3. Study limitations

The sample size of the study was relatively small for depicting an overall social representation of the German public. To offset this limitation, we controlled the samples as much as possible in terms of quotas. Moreover, expanded answer fields were deliberately provided to facilitate the collection of as many free word associations as possible.

Although participants were instructed to refrain from giving chain responses, we suspect that some participants did not always mentally return to the original stimulus.

Uncertainties were revealed at specific points of the inductive coding process. For example, many associations could have been coded into two or more categories. For the sake of clarity, double or more coding was performed within our study and on the networks. Associations such as “climate change” mainly triggered discussions between the two coders and were revisited even after assessing the terms for inter–coder–reliability. Since we wanted to maintain neutrality and coded associations with as little interpretation as possible, “climate change,” for example, could be classified as either “anthropogenic causes” or “change.” Thus, “climate change” was subordinated to the category “change” due to semantics. However, it should be noted that meaningful categorization requires knowledge of the association between a stimulus and its corresponding meaning (Lo Monaco et al., 2017). This requirement typically creates a challenge in interpreting the associations and is one of the most significant limitations. This task requires careful consideration of the context and meaning of the responses to determine a reliable and accurate social representation. In this type of analysis, associations are extracted from a broad context. Thus, the absence of contextualization hinders our comprehension of the intended meaning behind associations held by individuals. A potential solution was offered by Piermattéo et al. (2014), who recommended asking participants to write a sentence that expresses the meaning of their association in relation to the stimulus word—a technique referred to as “semantic contextualization” (Piermattéo et al., 2014; Lo Monaco et al., 2017).

Another difficulty was the creation of association networks. The clarity of the association network was highest for *biodiversity* when only the edges between associations occurring together at least three times were displayed; however, this filter setting was unsuitable for *biodiversity loss*. At the expense of uniformity, the edges between associations mentioned together twice were also visualized. In addition, we decided not to show all of the categories in color. Thus, in the present study, only the largest four categories are shown in color, plus an additional color for all other categories. We believed that this provided the network with a much clearer overview.

Association networks facilitate an overview of social representations but do not represent an individual network of a single participant. Moreover, due to time constraints, it was not possible to capture all associations for each individual. However, this limitation ensured that the associations were named as spontaneously as possible. Notably, in the brief time available, participants may not have been able to enter all of their associations in the fields provided.

Finally, the frequencies of word associations do not justify a direct inference of social representations (Wagner et al., 1996). However, the present study may provide a current indication of how the forms of understanding biodiversity and its loss are socially representative in Germany.

The Supplementary material contains the code book, original associations, and translations to make the process as transparent as possible and allow others to reconstruct our methodology for their own purposes. Despite these limitations, free word association tests remain a valuable tool for exploring social representations, provided the results are interpreted with these limitations in mind.

## 5. Conclusion

In the present study, social representations of the public regarding biodiversity and its loss were examined with the elicitation of free word associations and visualization via association networks. A process called “associative activation” underlies the events triggered when a participant views stimulus words. The elicited associations trigger many other associations in a spreading cascade of activity in the participant’s thought process. Each element is connected to other elements and supports and reinforces the others. A word might evoke memories, which may subsequently evoke emotions or other representations (Kahnemann, 2012).

Our results indicate that participants were able to express a complex understanding of biodiversity and its loss, encompassing various dimensions. The social representations surveyed reflected positive and negative dimensions, social dimensions, aspects of scientific work definition, and the role of climate change as a key driver of biodiversity loss. The concept of biodiversity and its loss appears to be anchored in people’s everyday practices and experiences. Thus, their social representations are presumably based on the shared knowledge, attitudes, and feelings that exist in German society. Previous studies measuring public understanding of biodiversity and its loss have focused on scientific terminology, revealing a lack of knowledge (Fischer and Young, 2007). These studies used a positivist approach that judged understanding as either “wrong” or “right.” However, the present study took a social representation theory perspective and revealed that the German public has diverse meanings for the stimulus words “biodiversity” and “biodiversity loss.” Understanding these representations is crucial for inferring the causal factors behind human thought processes and actions.

Furthermore, a novel approach to association networks that utilize the software for social network analysis was employed in the present study. Our association networks visualized the social representations described above and provided deeper insights into participants’ perceptions and understanding of biodiversity and its loss. By employing methods that determine how people organize their thinking about biodiversity and its loss, it may be possible to assess this relationship with social representations. By using association network analysis, we were able to identify which social representations were most central and select those that might hold the greatest potential for biodiversity conservation, outreach, and educational programs. However, creating more associations in the minds of the public should not be the primary focus. Rather, we suggest that communications about biodiversity and biodiversity loss should draw on existing ideas from the center or periphery of our network and embrace and strengthen the links between the network’s existing ideas.

In conclusion, we argue that the general public’s wide-ranging understanding of biodiversity and its loss should be recognized and incorporated into conservation management and further research on these concepts. Such initiatives may be needed to improve public support for biodiversity management; for example, to raise awareness of associations with invasive species and land use change, which are major causes of biodiversity loss that were underrepresented in this study. An adaptive understanding of representations of biodiversity and its loss may foster improved communication about biodiversity, conservation, and management measures (Buijs et al., 2008). Studying social representations may lead to improving a common understanding of biodiversity and its loss in the German public.

## Data availability statement

The original contributions presented in the study are included in the article/Supplementary material, further inquiries can be directed to the corresponding author.

## Ethics statement

Ethical review and approval was not required for the study on human participants in accordance with the local legislation and institutional requirements. The patients/participants provided their written informed consent to participate in this study.

## Author contributions

AE: conceptualization, investigation, writing – original draft, formal analysis, visualization, and software. FK: investigation, methodology, writing – original draft preparation, and software. KN: conceptualization, investigation, methodology, writing – original draft preparation, and software. SH: validation and data curation. FF: conceptualization, investigation, writing – review and editing, resources, supervision, and project administration. All authors contributed to the article and approved the submitted version.

## Funding

We acknowledge support by the Deutsche Forschungsgemeinschaft (DFG) and the Open Access Publishing Fund of Osnabrück University.

## Conflict of interest

The authors declare that the research was conducted in the absence of any commercial or financial relationships that could be construed as a potential conflict of interest.

## Publisher’s note

All claims expressed in this article are solely those of the authors and do not necessarily represent those of their affiliated organizations, or those of the publisher, the editors and the reviewers. Any product that may be evaluated in this article, or claim that may be made by its manufacturer, is not guaranteed or endorsed by the publisher.
