# Supplementary material for: Free word association analysis of German laypeople’s perception of biodiversity and its loss
Source: Front Psychol. 2023 Jun 28;14:1112182. doi: 10.3389/fpsyg.2023.1112182 (PMC10338174; doi:10.3389/fpsyg.2023.1112182)
Supplement: Supplementary file 2 [file Table_2.DOCX]

Supplementary Material

# Supplementary Tables

**Table I**

Questionnaire Sections *self-reported knowledge* and *free word association* test in German (original) and English

| **German** | **English** |
| --- | --- |
| ***Vorwissen^1^*** | ***Self-reported knowledge*** |
| War Ihnen der Begriff „Biologische Vielfalt“ vor dem Assoziationstest bekannt?  Antwortformat:  1 = Ich habe davon gehört, und weiß, was der Begriff bedeutet. 2 = Ich habe davon gehört, aber ich weiß nicht, was der Begriff bedeutet.  3 = Ich habe noch nie davon gehört. | Were you familiar with the term ‘biodiversity’ before the association test?  Response format:  1 = I have heard of it, and I know what the term means.  2 = I have of it, but I don’t know what the term means.  3 = I have never heard of it. |
| ***Freier Wort-Assoziationstest^2^*** | ***Free word associationtest*** |
| Vorstellungen zum Begriff "Biologische Vielfalt/Verlust biologischer Vielfalt"  Kurzbeschreibung des Assoziationstests  Bitte lesen die folgenden Informationen zum Assoziationstest aufmerksam durch!  Die folgende Aufgabe ist Teil einer Studie über verbales Verhalten und beinhaltet so genannte Wortassoziationen. Ihre Antworten werden nicht individuell, sondern kollektiv für die gesamte Stichprobe bewertet.  Ihre Antworten sind komplett anonym und wir würden Sie darum bitten, Ihre Assoziationen frei zu äußern. Es gibt keine schlechten oder falschen Antworten, also wählen Sie bitte Ihre Antworten nicht aus, sondern schreiben Sie sie spontan in der Reihenfolge auf, in der sie Ihnen einfallen. Im Prinzip ist die Aufgabe leicht und einfach zu lösen. Wir werden Ihnen im Folgenden genau einen Begriff präsentieren.  Beim Lesen des Begriffs denken Sie an andere damit verbundene Wörter (Gegenstände, Ideen, Themen usw.). Wir bitten Sie, so viele einzelne Antworten wie möglich zu verfassen und lose Phrasen oder Sätze zu vermeiden. Es ist wichtig, dass Sie bei Ihren Antworten immer den vorgegebenen Begriff berücksichtigen. Wenn beispielsweise der Begriff "Tisch" wäre und Ihre Antwort "Schreiben" lautete, müssen Sie sich bei den folgenden Antworten wieder auf "Tisch" beziehen. Kettenantworten wie beispielsweise "Schreiben, Stift, Tinte, Blau, Meer, Segel usw." sind zu vermeiden.  Bitte arbeiten Sie ohne Eile, aber geben Sie ihr Bestes, um uns so viele Antworten wie möglich zu geben. Für die Aufgabe bekommen sie insgesamt eine Minute Zeit. Der Fragebogen geht nach der Minute automatisch weiter. Zur Orientierung wird Ihnen oben auf der Seite ein 1-Minuten-Countdown angezeigt.  Welche Assoziationen kommen Ihnen bei dem Begriff "Biologische Vielfalt/Verlust biologischer Vielfalt" in den Sinn?  Bitte notiere Sie hier alle Begriffe, die Ihnen einfallen. Bitte nur ein Wort pro Zeile schreiben. | Ideas about the term "biodiversity/loss of biodiversity  Short description of the association test  Please read the following information about the association test carefully!  The following task is part of a study on verbal behavior and involves so-called word associations. Your answers will not be scored individually, but collectively for the entire sample.  Your answers are completely anonymous, and we would ask you to express your associations freely. There are no bad or wrong answers, so please do not select your answers, but write them down spontaneously in the order in which they occur to you. In principle, the task is easy and simple to solve. We will present you with exactly one term below.  As you read the term, think of other words associated with it (objects, ideas, topics, etc.). We ask you to write as many individual answers as possible and avoid loose phrases or sentences. It is important that you always consider the given term in your answers. For example, if the term would be "table" and your answer was "writing," you must refer back to "table" in subsequent answers. Chain answers such as "writing, pen, ink, blue, sea, sail, etc." are to be avoided.  Please work without hurry but do your best to give us as many answers as possible. You will be given a total of one minute for the task. The questionnaire will automatically continue after the minute. For your orientation, a 1-minute countdown is displayed at the top of the page.  What associations come to mind when you hear the term "biodiversity/loss of biodiversity"?  Please write down here any terms that come to mind. Please write only one word per line. |
| **Definiton: „Biologische Vielfalt“^3,4^**  Unter dem Begriff „**Biologische Vielfalt“**, wissenschaftlich auch als Biodiversität bezeichnet, werden die drei Teilbereiche (1) Artenvielfalt, (2) Ökosystemvielfalt und die (3) genetische Vielfalt zusammengefasst.  (1) Artenvielfalt: Vielfalt an Tier-, Pflanzen-, Pilz- und Bakterienarten, die auf der Erde existieren.  (2) Ökosystemvielfalt: Vielfalt von Ökosystemen und Lebensräumen, die sich aufgrund ihrer Artenzusammensetzung und Standorfaktoren voneinander unterscheiden, wie zum Beispiel Wälder, Wiesen oder Seen.  (3) Genetische Vielfalt: Vielfalt von Genen, Erbinformationen und Erbgut innerhalb einer Art, zum Beispiel bei unterschiedlichen Hunderassen oder Kohlsorten.  Wenn Sie die Definiton von biologischer Vielfalt aufmerksam gelesen und verstanden haben, klicken Sie bitte auf „Weiter“. | **Definition: “Biological diversity”**  The term **"biological diversity"**, scientifically also referred to as biodiversity, comprises the three sub-areas of (1) species diversity, (2) ecosystem diversity, and (3) genetic diversity.  (1) Species diversity: diversity of animal, plant, fungal, and bacterial species that exist on Earth.  (2) Ecosystem diversity: diversity of ecosystems and habitats that differ from each other based on their species composition and site factors, such as forests, grasslands, or lakes.  (3) Genetic diversity: Diversity of genes, hereditary information, and genetic material within a species, for example in different breeds of dogs or varieties of cabbage.  If you have read and understood the definition of biodiversity carefully, please click on “Next.” |

*Note.*

^1^ BMU [Federal Ministry for the Environment, Nature Conservation, Nuclear Safety, and Consumer Protection] and BfN [Federal Agency for Nature Conservation]. (2019). *Nature Awareness Study - Population survey on nature and biodiversity*.

^2^ Szalay, L. B., & Deese, J. (1978). Subjective Meaning and Culture: An Assessment through Word Associations. In American Anthropologist (Issue 2) Lawrence Erlbaum Associate, Inc. https://doi.org/10.1525/aa.1980.82.2.02a00390

^3^Swingland, I. R. (2001). *Biodiversity, Definition of*. In Encyclopedia of Biodiversity: Second Edition (pp. 377–391). Elsevier Inc. https://doi.org/10.1016/B978-0-12-384719-5.00009-5

^4^UN [United Nations Conference on Environment and Development]. (1992). Convention on Biological Diversity.

**Table II**

Associations with defined top 10 categories derived therefrom for *biodiversity* and *biodiversity loss*

| Associations | Definition | Category |
| --- | --- | --- |
|  | *All associations that describe …* |  |
| animal, insect, bird | … the totality of the eukaryotic organisms that live in the kingdom of the animals (Wetzel, 2020). | Animal |
| wrinkles, freedom | … something that cannot be assigned to any existing category; or cannot be clearly enough interpreted. | Other |
| plant, flower, tree | … characteristics of a plant, for example growth forms, functional pollination units or plant species (Cavalier-Smith, 1998). | Plant |
| diversity, variety | … diversity in general, diversity of a subject or species as well as structural diversity (Toepfer, 2011a). | Diversity |
| forest, lake, desert, | … ecosystems, biotopes, and habitat which act as habitat for e.g., animal and plants (Toepfer, 2011a, 2011b). | Habitat |
| extinct, species loss | … the decline of species in specific habitats or larger contexts (Swingland, 2001). | Species loss |
| forest decline, extinct | … the process of extinction or the irreversible state of extinct animals and plants (Duden, n.d.) | Animal and plant extinction |
| pesticides, eradication | … humans as the cause of the loss of biodiversity or human actions that lead to its loss (Slingenberg et al. 2009). | Anthropogenic causes |
| threatened, dreary | … the nature of someone or something like an object, nature, or a species (Duden, n.d.) | States |
| insect hotel, protection | … strategies to stop the loss of biodiversity, for example species protection, protection of habitats or organization of ecosystems (Cooke et al. 2013). | Biological Conservation |

*Note.* All references will be found at the end of the document.

**Table III**

Associations with defined categories derived therefrom for *biodiversity* and *biodiversity loss*

| **Associations** | **Definition** | **Category** |
| --- | --- | --- |
|  | *All associations that describe …* |  |
| nature, natural | … the physical characteristics and processes not of human origin (Hartig et al., 2014). | Nature |
| apple, egg | … for the purposes of the regulation, 'food' any substance or product intended to be, or reasonably expected to be ingested by humans in a processed, partially processed, or unprocessed state (Europäisches Parlament und Eurupäischer Rat, 2002) | Food |
| sad, greed | … an affectively experienced emotion or subjectively experienced feelings, such as satisfaction, sadness, fear, or anger (Colman, 2015). There are the following emotion categories: Interest, Joy, Sadness, Anger, Fear, Concern, Disgust, Contempt, Surprise, Happiness (Izard et al. 1974). | Affective/Emotion |
| generation, DNA | … characteristics of genetics. Genetics is the scientific study of heredity and variation (Rittner & McCabe, 2004). | Genetics |
| human, man | … characteristics of a human being like the upright gait, characteristics of gender (Carroll, 2003). | Human |
| grey, green | … a color. A color is a mode of appearance of things perceptible by the eye, based on the various reflection and absorption of light (Duden, n.d.). | Colors |
| economics, money | … characteristics of economy, such as the totality of facilities and measures that relate to the production and consumption of economic goods (Duden, n.d.). | Economic |
| monotonous, one-sided | … characteristics of similarities of things or situations. | One-sidedness |
| parc, zoo, museum | … the characteristics of cultural landscapes that, unlike natural landscapes, are influenced and modified by humans (Duden, n.d.). | Cultural landscape |
| ecology, food chain | … the study of interactions between organisms and between organisms and their abiotic environment (Markl et al., 2019). | Ecology |
| homosexual, reproduction | … the existence of different genders for the purpose of genetic exchange and related actions and other characteristics of sexual orientation (Markl et al., 2019). | Sexuality |
| creatures, sea creatures | … the ability to sustain and renew themselves, beings with organic life (Markl et al., 2019). | Creatures |
| climate change, change | … a processes and characteristics for processes in which a change is taking place | Change |
| flood, plague, disaster | … natural disasters, which are catastrophic events with atmospheric, geological, and hydrological causes (e.g., droughts, earthquakes, floods, hurricanes, landslides), these can result in fatalities, property damage, and environmental degradation (Xu et al., 2016). | Nature Disasters |
| agriculture, field, breeding | … economic activities in which land and livestock are involved as production factors alongside labor, capital and know-how, and whose central outputs are agricultural products (Duden, n.d.) | Agriculture |
| air, wind, climate | … inanimate as well as animate conditions of an organism's internal and external environment that affect its entire lifespan (Rittner & McCabe, 2004). | Environment |

*Note.* References will be found at the end of the document.

**Table IV**

Complete statistical network analysis for association networks of *biodiversity* and *biodiversity loss*

| **Biodiversity** | Degree  centrality | Betweenness  centrality | **Biodiversity loss** | Degree  Centrality | Betweenness  Centrality |
| --- | --- | --- | --- | --- | --- |
| animal | 46 | 0.281446 | species loss | 21 | 0.168551 |
| plant | 42 | 0.162218 | climate change | 21 | 0.122119 |
| nature | 33 | 0.048346 | plant | 18 | 0.082287 |
| human | 26 | 0.043076 | insect | 16 | 0.036083 |
| flower | 22 | 0.023524 | bee | 16 | 0.030996 |
| species diversity | 24 | 0.017981 | become extinct | 11 | 0.015697 |
| environment | 24 | 0.024316 | monoculture | 9 | 0.036608 |
| insect | 28 | 0.030997 | nature | 16 | 0.032286 |
| species | 19 | 0.01303 | bee extinction | 9 | 0.032333 |
| bird | 24 | 0.016977 | bird | 10 | 0.006659 |
| diversity | 18 | 0.014353 | pesticides | 9 | 0.018921 |
| species richness | 10 | 0.003519 | environmental pollution | 7 | 0.006609 |
| forest | 11 | 0.003993 | environment | 12 | 0.027465 |
| fauna | 15 | 0.008322 | species conservation | 6 | 0.004059 |
| ecosystem | 15 | 0.006018 | sad | 2 | 0.000626 |
| vegetable | 8 | 0.003653 | loss | 4 | 0.005327 |
| creature | 15 | 0.004053 | gene manipulation | 10 | 0.007689 |
| fruit | 7 | 0.003283 | forest fire | 7 | 0.004489 |
| tree | 12 | 0.006022 | sea | 8 | 0.0082 |
| environmental protection | 14 | 0.003908 | butterfly | 10 | 0.006018 |
| flora | 14 | 0.006383 | tree extinction | 2 | 0 |
| bee | 12 | 0.003435 | red list | 8 | 0.021453 |
| animal welfare | 6 | 0.000102 | animal | 14 | 0.011912 |
| extinct | 19 | 0.007637 | species diversity | 14 | 0.024859 |
| garden | 12 | 0.002397 | insect extinction | 5 | 0.007722 |
| life | 16 | 0.00393 | climate | 10 | 0.004802 |
| mammal | 15 | 0.004337 | eradication | 12 | 0.035709 |
| jungle | 15 | 0.005505 | animal life | 8 | 0.001921 |
| biotope | 12 | 0.004007 | bad | 7 | 0.007177 |
| variety | 9 | 0.00148 | human | 18 | 0.063247 |
| individual | 7 | 0.000355 | tree | 12 | 0.010103 |
| colorful | 10 | 0.001007 | vegetable | 1 | 0 |
| water | 9 | 0.0047 | unhealthy | 6 | 0.00758 |
| animal species | 7 | 0.000907 | monotony | 7 | 0.008789 |
| sea | 12 | 0.001648 | forest decline | 4 | 0.000884 |
| woman | 8 | 0.000408 |  |  |  |
| dog | 6 | 0.000233 |  |  |  |
| difference | 8 | 0.001213 |  |  |  |
| food | 4 | 0.000369 |  |  |  |
| fish | 13 | 0.007007 |  |  |  |
| sustainability | 10 | 0.000286 |  |  |  |
| egg | 5 | 0.001837 |  |  |  |
| waterbody | 13 | 0.002028 |  |  |  |
| nature protection | 11 | 0 |  |  |  |
| Darwin | 9 | 0.001784 |  |  |  |
| species loss | 8 | 0.000764 |  |  |  |
| species conservation | 10 | 0 |  |  |  |
| threatened | 12 | 0.002102 |  |  |  |
| man | 8 | 0.000408 |  |  |  |
| nature conservation | 11 | 0 |  |  |  |
| species protection | 10 | 0 |  |  |  |

*Note.* Results of degree centrality are not normalized.

All statistics were calculated in Gephi.

# Supplementary Figures

**Figure I**

Original Gephi network for *biodiversity* without any presetting


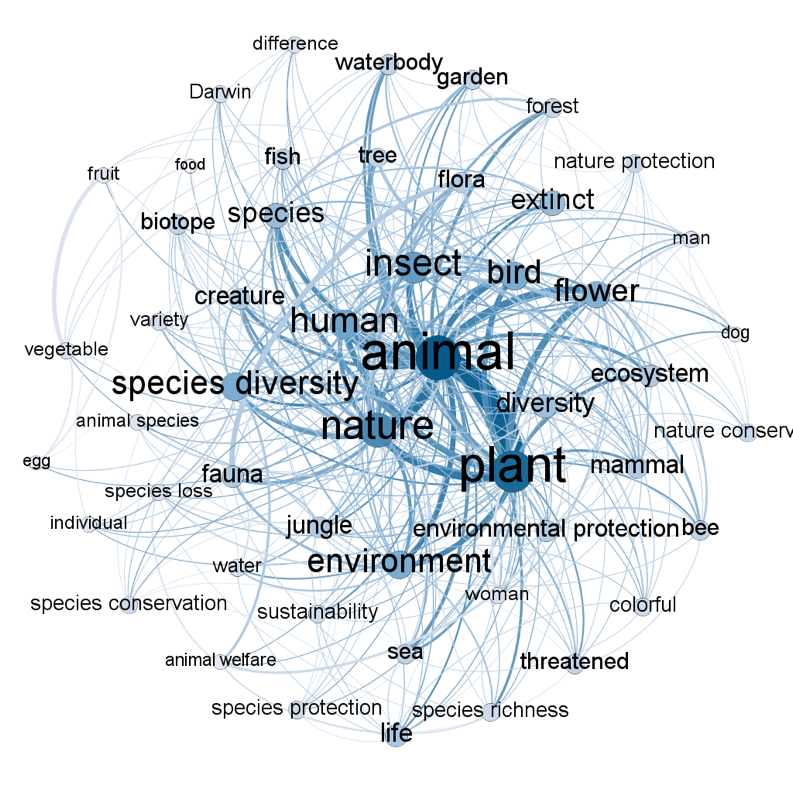


**Figure II**

Original Gephi network for *biodiversity loss* without any presetting


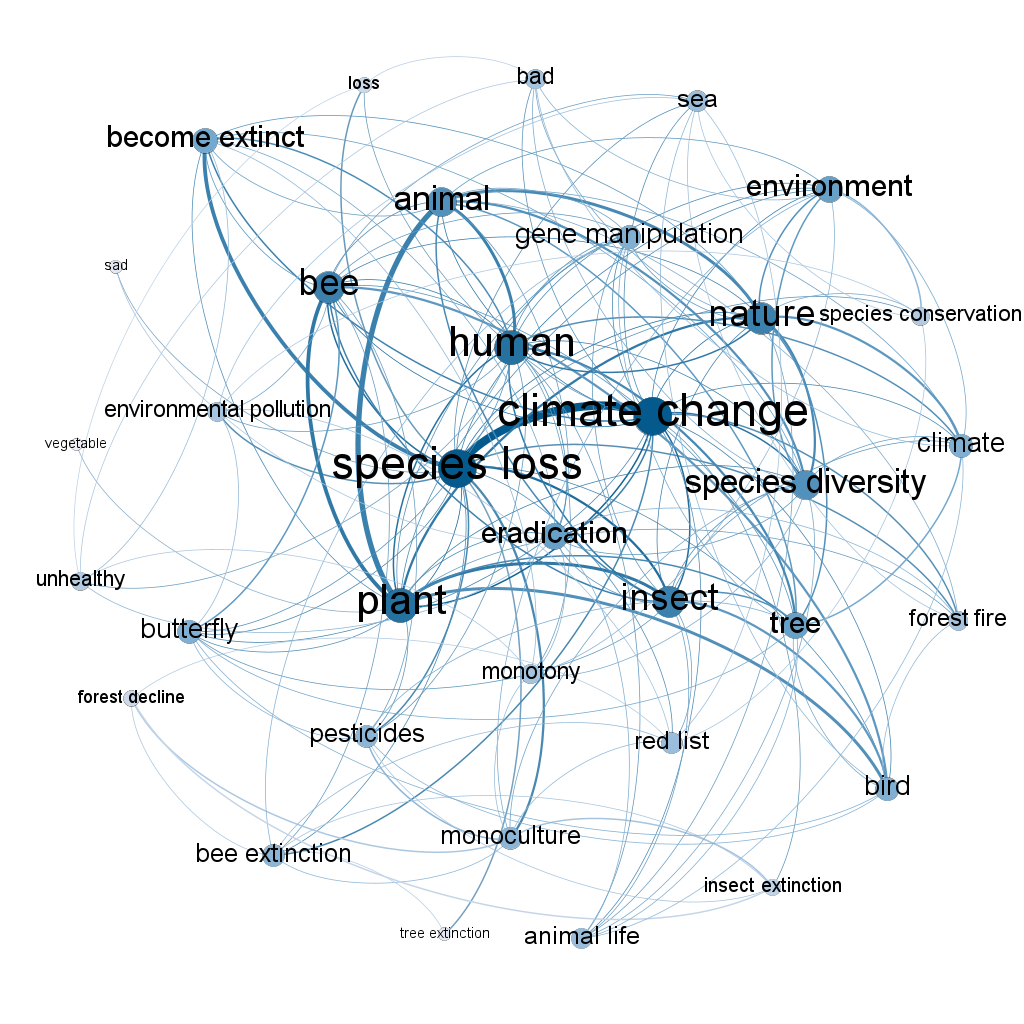


# References

Carroll, S. B. (2003). Genetics and the making of Homo sapiens. *Nature*, *422*, 849–857. https://doi.org/10.1038/nature01495

Cavalier-Smith, T. (1998). A revised six-kingdom system of life. *Biological Reviews*, *73*, 203– 266. https://doi.org/https://doi.org/10.1111/j.1469-185X.1998.tb00030.

Colman, A. J. (2015). *A dictionary of psychology*. Oxford University Press.

Cooke, S. J., Sack, L., Franklin, C. E., Farrell, A. P., Beardall, J., Wikelski, M., & Chown, S. L. (2013). What is conservation physiology? Perspectives on an increasingly integrated and essential science. *Conservation Physiology*, *1*(1). https://doi.org/10.1093/conphys/cot001

Dudenredaktion [Duden] (n.d.). *Duden Online. German Universal Dictionary [the comprehensive dictionary of meanings of the contemporary German language]*.

Europäische Kommission & Europäisches Parlament. (2002). *Verordnung (EG) Nr. 178/2002 (Lebensmittelbasisverordnung).*

Hartig, T., Mitchell, R., de Vries, S., & Frumkin, H. (2014). Nature and health. *Annual Review of Public Health*, *35*, 207–228. https://doi.org/10.1146/annurev-publhealth-032013- 182443

Izard, C. E. (1977). *Human Emotions*. Springer US. https://doi.org/10.1007/978-1-4899-2209- 0

Rittner, D., & McCabe, T. (2004). *Encyclopedia of Biology*. Facts On File.

Markl, J., Sadava, D., Hillis, D., Craig Heller, H., & Hacker, S. (2019). *Purves Biologie*. https://link.springer.com/content/pdf/10.1007/978-3-662-58172-8.pdf

Toepfer, G. (2011a). Historical dictionary of biology. In *Historical Dictionary of Biology* (Vol. 2). J.B. Metzler. https://doi.org/10.1007/978-3-476-00455-0

Toepfer, G. (2011b). Historical dictionary of biology. In *Historical Dictionary of Biology* (Vol. 1). J.B. Metzler. https://doi.org/10.1007/978-3-476-00439-0

Schmid, T., & Pröll, E. (2019). *Umwelt-und Bioressourcen-management für eine nachhaltige Zukunftsgestaltung*. https://doi.org/https://doi.org/10.1007/978-3-662-60435-9

Slingenberg, A., Braat, L.C., Windt, H.V., Rademaekers, K., Eichler, L., & Turner, K. (2009). *Study on understanding the causes of biodiversity loss and the policy assessment framework.* European Commission.

Swingland, I. R. (2001). Biodiversity, Definition of. In *Encyclopedia of Biodiversity: Second Edition* (pp. 377–391). Elsevier Inc. https://doi.org/10.1016/B978-0-12-384719-5.00009-5

Wetzel, K. (2020). Unterschiede zwischen Pflanze und Tier. In *Grundriss der allgemeinen Botanik* (pp. 3–4). DeGruyter.

Xu, J., Wang, Z., Shen, F., Ouyang, C., & Tu, Y. (2016). Natural disasters and social conflict: A systematic literature review. In *International Journal of Disaster Risk Reduction* (Vol. 17, pp. 38–48). Elsevier Ltd. https://doi.org/10.1016/j.ijdrr.2016.04.00
